# Two new species of *Campanella* and *Crustomyces* (*Agaricales*, *Basidiomycota*) from China

**DOI:** 10.3897/mycokeys.138.203655

**Published:** 2026-08-04

**Authors:** Wanting Liu, Yinglian Deng, Xiangfu Liu, Changlin Zhao

**Affiliations:** 1 Key Laboratory of Forest Resources Conservation and Utilization in the Southwest Mountains of China Ministry of Education, Yunnan Provincial Key Laboratory for Conservation and Utilization of In-forest Resource, Southwest Forestry University, Yunnan, Kunming, 650224, China Department Microbial Drugs (MWIS), Helmholtz-Centre for Infection Research Braunschweig Germany; 2 College of Forestry, Southwest Forestry University, Kunming 650224, China College of Forestry, Southwest Forestry University Kunming China; 3 Modern Industry School of Edible-fungi, Southwest Forestry University, Kunming 650224, China Key Laboratory of Forest Resources Conservation and Utilization in the Southwest Mountains of China Ministry of Education, Yunnan Provincial Key Laboratory for Conservation and Utilization of In-forest Resource, Southwest Forestry University Kunming China; 4 Department Microbial Drugs (MWIS), Helmholtz-Centre for Infection Research, 38124 Braunschweig, Germany Modern Industry School of Edible-fungi, Southwest Forestry University Kunming China

**Keywords:** Biodiversity, taxonomy, wood-inhabiting fungi, Yunnan Province

## Abstract

During a survey of *Agaricales* fungi in tropical regions of China, two new species within this order from Yunnan Province were delineated based on comprehensive morphological and phylogenetic investigations. Morphologically, *Campanella albomarginata* is distinguished by its resupinate, coriaceous basidiomata with a white to cream hymenial surface, a monomitic hyphal system with clamped generative hyphae, subcylindrical basidia with four sterigmata, and drop-shaped basidiospores (9.5–12 × 5–7.5 µm). *Crustomyces odontoideus* is characterized by an odontioid hymenial surface, a monomitic hyphal system with clamped generative hyphae, subcylindrical to subclavate basidia with four sterigmata, and ovoid to broadly ellipsoid basidiospores measuring 4–4.5 × 2.5–3 µm. Phylogenetic analyses based on a two-marker dataset (ITS and LSU) support the recognition of both taxa as distinct species belonging to *Campanella (Campanellaceae)* and *Crustomyces (Cystostereaceae)*, respectively, within *Agaricales*. Descriptions, illustrations, and phylogenetic analyses of the new species are provided. The results augment knowledge of the species diversity and taxonomy of fungi in southwestern China.

## Introduction

The order *Agaricales* was erected by Chevallier (1826), with *Agaricaceae* Chevall. as the type family, and is one of the fungal orders that includes wood-inhabiting fungi within *Agaricomycetes*, *Basidiomycota* (Larsson and Larsson 2003; Vizzini et al. 2024; Heitman 2025; Hibbett et al. 2025; Niskanen et al. 2026). Its basidiomata exhibit a remarkable spectrum of morphological diversity, ranging from resupinate (corticioid) to stereoid, clavarioid, agaricoid, or highly complex forms, with lamellate, smooth, wrinkled, odontoid, or poroid hymenophores (James et al. 2020; Spirin et al. 2024; Zhang et al. 2024; Guan et al. 2025; Wiens et al. 2026). Fungi of this order occur in a wide range of terrestrial habitats, e.g., forests, grasslands, and wetlands (Frank et al. 2010; Guan et al. 2025). More than 40,000 species have been assigned to *Agaricales* (Dong et al. 2025), which comprises 560 genera distributed across 62 families within 12 suborders (Hyde et al. 2024; Oliveira et al. 2024; Vizzini et al. 2024).

The genus *Campanella* Henn. (*Campanellaceae*) was introduced by Hennings (1895) and was typified by *Ca. buettneri* Henn. (Horak 1986; Matheny et al. 2006). A comprehensive study on the taxonomy of *Marasmiaceae* s.l. revealed that the genera *Brunneocorticium* Sheng H. Wu, *Campanella*, *Neocampanella* Nakasone, Hibbett & Goranova, *Tetrapyrgos*, and the *Marasmiellus candidus* (Fr.) Singer group represented a distinct lineage separate from *Marasmiaceae* s.str. and *Omphalotaceae* Bresinsky, and this lineage was circumscribed as *Campanellaceae* (Oliveira et al. 2024). Based on both morphology and phylogenetic analyses, the taxonomy of wood-inhabiting *Agaricales* was revised, and the new species *Ca. yunnanensis* J.H. Dong & C.L. Zhao was reported in *Campanella* (Dong et al. 2025). Species of *Campanella* are characterized by small, lignicolous, pleurotoid to resupinate basidiomata with a distinctive hymenophore composed of vein-like or ridge-like anastomosing lamellae and smooth, colorless, inamyloid basidiospores (Singer 1975; Farook and Manimohan 2014; Dai 2022; Dong et al. 2025). Many species of *Campanella* have been transferred to other genera, such as *Arrhenia* Fr., *Gloiocephala* Massee, *Metacampanella* R.H. Petersen, *Marasmiellus* Murrill, *Resupinatus* Nees ex Gray, and *Rhodoarrhenia* Singer (Petersen and Hughes 2025). The genus *Campanella* currently contains 64 specific names registered in Index Fungorum, but the accepted number of species is 42 (Desjardin et al. 2017; Dong et al. 2025).

*Cystostereaceae* Jülich is a small family of wood-decaying fungi in *Agaricales*, composed of seven genera: *Cericium* Hjortstam, *Crustomyces* Jülich, *Cystidiodontia* Hjortstam, *Cystostereum* Pouzar, *Parvobasidium* Jülich, *Parvodontia* Hjortstam & Ryvarden, and *Rigidotubus* J.Song, Y.C.Dai & B.K.Cui (Pouzar 1959; Jülich 1978, 1981; Larsson 2007; Song et al. 2018; Dong et al. 2025). *Crustomyces* was established by Jülich (1979), with *Cr. subabruptus* (Bourd. & Galzin) Jülich as the type species. The generic concept of *Crustomyces* was revised to include a monomitic hyphal system, a poroid hymenophore, and thick-walled basidiospores (Li et al. 2022). Originally, two species were included in the genus, viz., *Cr. subabruptus* and *Cr. pini-canadensis* (Schwein.) Jülich (Jülich 1978). Recently, three new combinations, *Cr. isabellinus* (Berk. & Broome) Yue Li, Nakasone & S.H. He, *Cr. laminiferus* (Berk. & M.A. Curtis) Yue Li, Nakasone & S.H. He, and *Cr. tephroleucus* (J. Song, Y.C. Dai & B.K. Cui) Yue Li, Nakasone & S.H. He, were proposed, and a new species, *Cr. subisabellinus* Shun Liu & Biao Zhu, was described (Li et al. 2022; Liu et al. 2025). According to the MycoBank database (http://www.MycoBank.org, accessed on 9 July 2026) and Index Fungorum (http://www.indexfungorum.org, accessed on 9 July 2026), 16 species of *Crustomyces* have been registered, but 13 species are accepted, and 10 species belonging to this genus occur in China, namely, *Cr. albidus* Yue Li, Nakasone, C.L. Zhao & S.H. He, *Cr. indecorus* Hjortstam, *Cr. isabellinus*, *Cr. juniperi* S.L. Liu & L.W. Zhou, *Cr. laminiferus*, *Cr. pini-canadensis*, *Cr. scytinostromoides* S.L. Liu & L.W. Zhou, *Cr. subabruptus* (Bourdot & Galzin) Jülich, *Cr. subisabellinus*, and *Cr. tephroleucus* (Li et al. 2022; Miettinen et al. 2023; Liu et al. 2025).

Currently, molecular phylogenetic analyses based on multiple genes play a vital role in the taxonomy of *Agaricales*, not only in the delineation of new species but also in revising the classification of currently unclassified taxa (Dong et al. 2025; Tarafder et al. 2026). In recent years, the species diversity of resupinate *Agaricales* has been described or better defined using morphological and molecular analyses, and the results have revealed the hidden diversity of this group and of several corticioid genera (Dong et al. 2025; Niskanen et al. 2026; Zhuang et al. 2026). Despite the increasing attention to resupinate *Agaricales*, a large number of potentially new species remain hidden in underexplored regions, particularly in tropical and subtropical Asia, awaiting further taxonomic and phylogenetic assessment.

During investigations of *Agaricales* fungi in Yunnan Province, China, two potentially new species were collected. To clarify the placement and relationships of these specimens, a phylogenetic and taxonomic study based on ITS and LSU sequences was conducted. These specimens were assigned to the genera *Campanella* and *Crustomyces* of *Agaricales*.

## Materials and methods

### Sample collection and herbarium specimen preparation

Fresh basidiomata were collected from the Dehong region in Yunnan Province, China. The samples were photographed *in situ*, and fresh macroscopic details were recorded. Photographs were taken using a Nikon D7100 camera. The specimens were transported to a field station, where the fruiting bodies were dried using an electronic food dryer at 45 °C (Dong et al. 2024). Once dried, the specimens were sealed in envelopes and zip-lock plastic bags and labeled. The dried specimens were deposited in the herbarium of Southwest Forestry University (SWFC), Kunming, Yunnan Province, China.

### Morphology

Morphological characterization was performed based on field observations and the examination of dried specimens, following earlier taxonomic studies (Carpouron et al. 2024; Dong et al. 2025). Micromorphological characters were examined and illustrated using dried voucher specimens under a light microscope, according to the methods described by Deng et al. (2026). Microscopic observations were carried out at magnifications of up to 1000× using a Nikon Eclipse 80i microscope (Nikon, Tokyo, Japan). The following abbreviations were used: KOH = 5% aqueous potassium hydroxide solution; CB+ = cyanophilous; CB = cotton blue; CB– = acyanophilous; IKI = Melzer’s reagent; IKI– = both inamyloid and indextrinoid; *L* = mean spore length (arithmetic average of all spores); *W* = mean spore width (arithmetic average of all spores); *Q* = variation in the *L*/*W* ratios among the specimens studied; and *n* = *a*/*b* (number of spores [*a*] measured from a given number [*b*] of specimens).

### DNA extraction, amplification, and sequencing

The CTAB Rapid Plant Genome Extraction Kit-DN14 (Aidlab Biotechnologies Co., Ltd., Beijing) was used to obtain genomic DNA from dried specimens following the procedure of a previous study (Wang et al. 2026). The ITS region was amplified using the primer pair ITS5 and ITS4 (White et al. 1990). The nuclear LSU region was amplified using the primer pair LR0R and LR7 (Vilgalys and Hester 1990). The PCR procedure for ITS was as follows: initial denaturation at 95 °C for 3 min; 35 cycles at 94 °C for 40 s, 54 °C for 45 s, and 72 °C for 1 min; and a final extension at 72 °C for 10 min. The PCR procedure for LSU was as follows: initial denaturation at 94 °C for 1 min; 35 cycles at 94 °C for 30 s, 50 °C for 1 min, and 72 °C for 1.5 min; and a final extension at 72 °C for 10 min. The PCR products were purified and sequenced at Kunming Tsingke Biological Technology Limited Company, Kunming, Yunnan Province, P.R. China. All newly generated sequences were deposited in GenBank (Table 1).

**Table 1.** Names, voucher numbers, references, and corresponding GenBank accession numbers of the taxa used in the phylogenetic analyses. The asterisk (*) indicates the type specimen; the dash (—) indicates an unavailable sequence.

**Species name**	**Voucher**	**Locality**	**GenBank accession**		**Reference**
			**ITS**	**LSU**	
*Amanita brunnescens*	AFTOL ID 673	USA	AY789079	AY631902	Matheny et al. (2006)
*Amanita subglobosa*	HKAS:58837	China	JN943177	JN941152	Vizzini et al. (2024)
*Amparoina heteracantha*	HTBM2964	China	PX308916	—	Yang et al. (2025)
*Amylocorticium cebennense*	CFMR: HHB-2808	USA	GU187505	GU187561	Vizzini et al. (2024)
*Anthracophyllum archeri*	AFTOL ID 973	USA	DQ404387	AY745709	Matheny et al. (2006)
*Aphanobasidium pseudotsugae*	HHB-822	USA	GU187509	GU187567	Larsson (2007)
*Aphanobasidium pseudotsugae*	UC 2023153	Sweden	KP814353	AY586696	Larsson (2007)
*Apioperdon pyriforme*	AFTOL ID 480	USA	AY854075	AF287873	Vizzini et al. (2024)
*Asterophora lycoperdoides*	CBS170.86	Switzerland	AF357037	AF223190	Matheny et al. (2006)
*Bolbitius vitellinus*	AFTOL ID 730	USA	DQ200920	AY691807	Matheny et al. (2006)
*Camarophyllopsis hymenocephala*	AFTOL ID 1892	USA	DQ484066	DQ457679	Matheny et al. (2006)
*Campanella albomarginata*	CLZhao 42114 *	China	PZ244091	PZ253250	Present study
*Campanella albomarginata*	CLZhao 48992	China	—	PZ253251	Present study
*Campanella bispora*	F:C0004046F (M-296)	India	PX956804	—	Farook and Manimohan (2014)
*Campanella buettneri*	DED 8276 (SFSU)	USA	MF075136	MF075138	Desjardin and Perry (2017)
*Campanella burkei*	SFSU: BAP 632 *	USA	MF100970	—	Desjardin and Perry (2017)
*Campanella candida*	PDD: 102184	New Zealand	OQ282823	—	Unpublished
*Campanella gregaria*	PERTH *	Australia	—	MK277680	Boucher (2007)
*Campanella keralensis*	AF 342	India	MW462889	—	Desjardin and Perry (2017)
*Campanella pustulata*	SMF2382	Australia	JX444164	—	Lloyd (1919)
*Campanella pustulata*	AQ793972	Australia	JX444168	—	Lloyd (1919)
*Campanella subgregaria*	Liu 582*	China	PQ638413	PQ637011	Liu et al. (2025)
*Campanella subgregaria*	Liu 573	China	PQ638414	PQ637012	Liu et al. (2025)
*Campanella tristis*	JAC17081	New Zealand	PP407516	—	Segedin (1993)
*Campanella tristis*	PDD88322	New Zealand	PP407517	—	Segedin (1993)
*Campanella yunnanensis*	CLZhao 33866 *	China	PV197911	—	Dong et al. (2025)
*Campanella yunnanensis*	CLZhao 33868	China	PV197912	—	Dong et al. (2025)
*Candolleomyces striginus*	HKAS150762 (HT) *	China	PX308934	PX309050	Yang et al. (2025)
*Candolleomyces vagabundoides*	HKAS150768 (HT) *	China	PX308889	PX309007	Yang et al. (2025)
*Catathelasma ventricosum*	DAOM: 225247	Spain	MN017537	MN017477	Vizzini et al. (2024)
*Cericium gloeocystidiatum*	He 4332 *	China	—	MW557949	Zhang et al. (2024)
*Cericium gloeocystidiatum*	He 4728	China	—	MW557950	Zhang et al. (2024)
*Cheimonophyllum candidissimum*	AFTOL ID 1765	USA	DQ486687	DQ457654	Matheny et al. (2006)
*Chondrostereum purpureum*	AFTOL ID 441	USA	DQ200929	AF518607	Matheny et al. (2006)
*Chromocyphella lamellata*	AH 45802 *	Spain	MF623832	MF623831	Moreno et al. (2017)
*Chromocyphella meloana*	LIP GG-201018-003	Spain	ON059375	ON059179	Gruhn et al. (2023)
*Chromocyphella muscicola*	ARAN-Fungi 3324	Spain	MF623834	MF623834	Moreno et al. (2017)
*Clavaria zollingeri*	AFTOL ID 563	USA	AY854071	AY639882	Matheny et al. (2006)
*Clavulinopsis laeticolor*	AFTOL ID 984	USA	DQ202267	AY745693	Matheny et al. (2006)
*Collybiopsis melanopus*	SFSU: A.W. Wilson 54	Indonesia	NR137539	NG 060624	Wilson et al. (2004)
*Collybiopsis mesoamericana*	TENN 058613	Costa Rica	NR119583	KY019632	Schoch et al. (2014)
*Collybiopsis minor*	TENN F 059993 *	USA	MN413334	MW396880	Petersen and Hughes (2021)
*Cortinarius aurilicis*	TSJ1998-101	Denmark	DQ083772	AY684152	Frøslev et al. (2005)
*Cortinarius iodes*	IB19850061	USA	AF389133	AY702013	Matheny et al. (2006)
*Cortinarius violaceus*	AFTOL ID 814	USA	DQ486695	DQ457662	Matheny et al. (2006)
*Crinipellis birhizomorpha*	BRNM751593 *	Korea	KF380831	KF380835	Antonín et al. (2014)
*Crinipellis setipes*	Bandala4031	Mexico	JF930641	JF930642	Antonín et al. (2014)
*Crustomyces albidus*	He 6164 *	China	ON117191	ON117175	Li et al. (2022)
*Crustomyces albidus*	CLZhao 6194	China	MK404325	—	Li et al. (2022)
*Crustomyces isabellinus*	Yuan 12642	Vietnam	ON117185	ON117170	Li et al. (2022)
*Crustomyces juniperi*	LWZ 20180905-5 *	China	OR557241	OR527267	Liu et al. (2025)
*Crustomyces juniperi*	LWZ 20180905-3	China	OR557243	OR527269	Liu et al. (2025)
*Crustomyces laminiferus*	KHL 13057	Costa Rica	EU118622	EU118622	Larsson (2007)
*Crustomyces laminiferus*	TF 10	Nigeria	MH625705	MH625705	Li et al. (2022)
*Crustomyces odontoideus*	CLZhao 44314 *	China	PZ244092	PZ253252	Present study
*Crustomyces odontoideus*	CLZhao 44476	China	PZ244093	PZ253253	Present study
*Crustomyces scytinostromoides*	LWZ 20190808-14b *	China	OR557244	OR527270	Liu et al. (2025)
*Crustomyces scytinostromoides*	LWZ 20190807-15b	China	OR557245	OR527271	Liu et al. (2025)
*Crustomyces subabruptus*	KM 249975	UK	MZ159685	—	Li et al. (2022)
*Crustomyces subabruptus*	PC 0706577	France	MF183941	—	Li et al. (2022)
*Crustomyces subisabellinus*	Liu 907	China	PQ638408	PQ637007	Liu et al. (2025)
*Crustomyces subisabellinus*	Liu 937 *	China	PQ638407	PQ637006	Liu et al. (2025)
*Crustomyces tephroleucus*	Cui 13653	China	MF290418	MF290416	Song et al. (2018)
*Crustomyces tephroleucus*	Cui 13717 *	China	MF290417	MF290415	Song et al. (2018)
*Cystostereum crassisporum*	He 3995	China	—	ON117178	Li et al. (2022)
*Cystostereum crassisporum*	He 4740 *	China	ON117193	ON117179	Li et al. (2022)
*Cystostereum murrayi*	CBS:413.34	Germany	MH855588	MH867098	Vu et al. (2019)
*Cystostereum murrayi*	KHL 12496	Sweden	EU118623	EU118623	Larsson (2007)
*Cystostereum murrayi*	CBS 257.73	Germany	—	MH878328	Vu et al. (2019)
*Cystostereum submurrayi*	He 4379	China	ON117192	ON117177	Li et al. (2022)
*Cystostereum submurrayi*	WEI 18-033	China	OM942662	OM919711	Li et al. (2022)
*Effusomyces thailandicus*	He 4055c *	Thailand	ON117194	ON117180	Li et al. (2022)
*Effusomyces thailandicus*	He 4126	Thailand	—	ON117181	Li et al. (2022)
*Flammulina velutipes*	AFTOL ID 558	USA	AY854073	AY639883	Matheny et al. (2006)
*Gracilihypha abeliae*	CLZhao 21356 *	China	PP819704	PP826256	Yang et al. (2025)
*Gracilihypha abeliae*	CLZhao 21485	China	PP819706	PP826258	Yang et al. (2025)
*Gymnopus bicolor*	AWW116 *	USA	AY263423	AY639411	Wilson et al. (2004)
*Gymnopus dryophilus*	AFTOL-ID 559	USA	DQ241781	AY640619	Matheny et al. (2006)
*Hohenbuehelia atrocoerulea*	AMB: 18080 *	Italy	KU355304	KU355389	Vizzini et al. (2024)
*Hohenbuehelia tremula*	DAOM: 180808	Italy	KU355357	KU355405	Vizzini et al. (2024)
*Hydnangium carneum*	Trappe 31123	USA	KU685741	KU685892	Vizzini et al. (2024)
*Laccaria ochropurpurea*	AFTOL-ID 477	USA	AF006598	AY700200	Matheny et al. (2006)
*Limnoperdon incarnatum*	IFO: 30398	USA	DQ097363	AF426958	Matheny et al. (2006)
*Macrolepiota dolichaula*	AFTOL ID 481	USA	DQ22111	DQ411537	Vizzini et al. (2024)
*Macrotyphula juncea*	IO.14.177	Spain	MT232353	MT232306	Vizzini et al. (2024)
*Marasmius acerosus*	TYS458	Canada	OR636634	OR656952	Oliveira et al. (2024)
*Marasmius albopurpureus*	N.K.Zeng 2253	China	MT822924	MT829107	Dong et al. (2025)
*Moniliophthora aurantiaca*	UTC 253824	USA	JN692482	JN692483	Antonín et al. (2014)
*Moniliophthora perniciosa*	DIS71	Brazil	AY317136	AY916738	Matheny et al. (2006)
*Moniliophthora roreri*	C21	USA	AY916746	AY916744	Antonín et al. (2014)
*Mythicomyces corneipes*	AFTOL ID 972	USA	DQ404393	AY745707	Matheny et al. (2006)
*Nia vibrissa*	AH201905273	Japan	LC769783	LC769838	Nakagiri et al. 2024
*Omphalotus olearius*	AFTOL ID 1718	USA	DQ494681	DQ470816	Matheny et al. (2006)
*Omphalotus olivascens*	VT455	Austria	AF525063	AF261325	Matheny et al. (2006)
*Parvodontia austrosinensis*	He 4339	China	—	ON117182	Li et al. (2022)
*Parvodontia luteocystidia*	FP -102806	USA	OP935224	OP995724	Paez et al. (2024)
*Pleurotus dryinus*	AMB: 18868	Spain	OR863471	OR863538	Vizzini et al. (2024)
*Pleurotus fuscosquamulosus*	A. Baglivo 13-07-2014	Spain	OR863472	—	Vizzini et al. (2024)
*Pluteus romellii*	AFTOL ID 625	USA	AY854065	AY634279	Matheny et al. (2006)
*Pluteus variabilicolor*	AMB: 18872	Spain	OR863475	OR863541	Matheny et al. (2006)
*Porotheleum fimbriatum*	AFTOL ID 1725	USA	DQ490626	DQ457673	Vizzini et al. (2024)
*Porotheleum omphaliiforme*	AMB 18842	Spain	OM422773	OM423650	Consiglio et al. (2021)
*Pulverulina ulmicola*	TENN 029208 *	USA	NR119887	HQ179668	Consiglio et al. (2021)
*Radulomyces confluens*	Cui 5977	China	KU535661	KU535669	Zhao et al. (2016)
*Radulomyces confluens*	He 2224	China	KU535662	KU535670	Zhao et al. (2016)
*Radulotubus resupinatus*	Cui 8383 *	China	KU535660	KU535668	Zhao et al. (2016)
*Radulotubus resupinatus*	Dai 15315	China	KU535657	KU535666	Zhao et al. (2016)
*Rhodocollybia maculata*	AFTOL ID 540	USA	—	AY639880	Matheny et al. (2006)
*Sarcomyxa edulis*	HKAS 118864	China	OQ274994	OQ286065	Cai et al. (2023)
*Sarcomyxa serotina*	AFTOL-ID 536	USA	DQ494695	AY691887	Matheny et al. (2006)
*Schizophyllum commune*	DSH96 026	USA	AF249390	AF334751	Matheny et al. (2006)
*Schizophyllum radiatum*	AFTOL ID 516	USA	AY571060	AY571023	Matheny et al. (2006)
*Stagnicola perplexa*	ALV16926	Spain	MK351606	MK353790	Vizzini et al. (2019)
*Tortoperdon suspectum*	HKAS150757 (HT) *	China	PX308896	PX309010	Yang et al. (2025)
*Tortoperdon suspectum*	HTBM 0362	China	PX308892	PX309012	Yang et al. (2025)
*Tricholoma aestuans*	AFTOL ID 497	USA	DQ494699	AY700197	Matheny et al. (2006)
*Tricholoma myomyces*	KMS 589	USA	DQ825428	U76459	Matheny et al. (2006)
*Xanthagaricus popcorneus*	HKAS 150767 (HT) *	China	PX308888	PX309006	Yang et al. (2025)
*Xanthagaricus popcorneus*	HTBM 2744	China	PX308886	PX309005	Yang et al. (2025)
*Xerophorus dominicanus*	127428 (JBSD) *	Spain	MN017550	MN017489	Vizzini et al. (2020)
*Xerophorus donadinii*	18222 (AMB)	Spain	MN017551	MN017490	Vizzini et al. (2020)
*Xuaniella urbica*	HKAS 150761 (HT) *	China	PX308937	PX309053	Yang et al. (2025)

### Phylogenetic analyses

Phylogenetic analyses followed the methods described by Dissanayake et al. (2020). Newly generated sequence data were initially subjected to a BLAST search at NCBI to identify the most probable closely related taxa in GenBank (http://blast.ncbi.nlm.nih.gov). Sequence data were retrieved from GenBank based on recent publications (www.ncbi.nlm.nih.gov/nuccore). The sequences were aligned using MAFFT version 7 (https://mafft.cbrc.jp/alignment/server/; Katoh et al. 2019) with the G-INS-I strategy. The alignment was manually adjusted using AliView version 1.27 (Larsson 2014). The datasets were initially aligned, and the ITS and LSU sequences were subsequently combined using Mesquite version 3.51. FASTA files were converted to NEXUS format using the online tool available on the ALTER website (http://sing.ei.uvigo.es/ALTER/; Glez-Peï et al. 2010). Phylogenetic trees were constructed based on randomized accelerated maximum likelihood (ML) and Bayesian inference (BI) analyses following previously established procedures (Wijesinghe et al. 2025; Deng et al. 2026).

**Figure 1.**
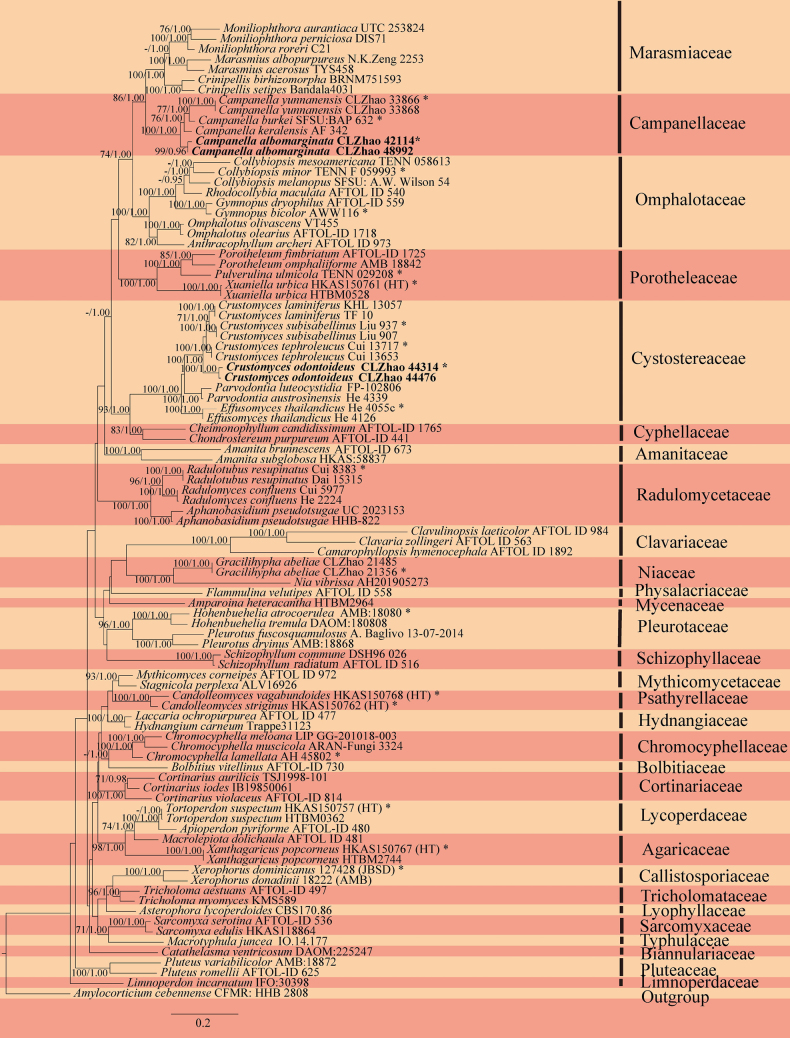
Phylogram generated from a maximum likelihood analysis of the two new species and related species in *Agaricales* based on combined ITS and LSU sequence data; branches are labeled with maximum likelihood bootstrap values ≥ 70% and Bayesian posterior probabilities ≥ 0.95. The new species are in bold.

**Figure 2.**
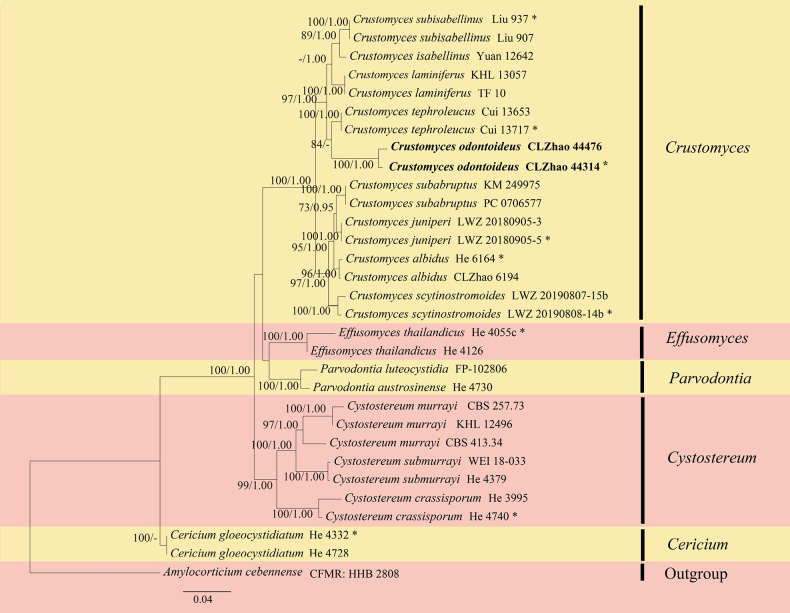
Phylogram generated from a maximum likelihood analysis of *Crustomyces odontoideus* and related species in *Cystostereaceae* based on combined ITS and LSU sequence data; branches are labeled with maximum likelihood bootstrap values ≥ 70% and Bayesian posterior probabilities ≥ 0.95. The new species is in bold.

Maximum likelihood analysis was performed using the CIPRES Science Gateway (http://phylo.org/portal2/login!input.action; Miller et al. 2012) based on the dataset using the RAxML-HPC BlackBox tool, with RAxML set to halt bootstrapping automatically and the maximum runtime set to 0.25 h; the best tree was obtained using an ML search. Other parameters in the ML analysis were set to their defaults, and statistical support values were obtained using nonparametric bootstrapping with 1,000 replicates. Bayesian inference analysis was performed on the dataset using MrBayes v3.2.7a (Ronquist et al. 2012). BI was conducted for 7,000,000 and 800,000 generations for Figs 1, 2, respectively, with two independent runs, each consisting of four Markov chains (one cold and three heated). Trees were sampled every 1,000 generations, yielding a total of 2,000 trees. The first 25% of the trees (500 trees) were discarded as burn-in, and the posterior distribution was estimated based on the remaining 1,500 trees. The burn-in fraction of 25% was determined by inspecting likelihood convergence and the stationarity of parameter traces in MrBayes v3.2.6. Convergence between runs was further assessed by ensuring that the average standard deviation of split frequencies was below 0.01.

Phylogenetic trees were visualized and adjusted using FigTree v1.4.0 (http://tree.bio.ed.ac.uk/software/figtree), and the exported files were edited using Adobe Illustrator CS6 software (Adobe Systems, USA). Branches of the consensus tree with ML bootstrap support ≥ 70% and Bayesian posterior probabilities ≥ 0.95 were considered strongly supported.

### Genealogical concordance phylogenetic species recognition (GCPSR) analysis

Genealogical concordance phylogenetic species recognition (GCPSR) analysis was employed to detect significant recombination events (Quaedvlieg et al. 2014). The data were analyzed using the pairwise homoplasy index (PHI) test in SplitsTree 4 to determine the level of recombination among closely related species (Bruen et al. 2006; Huson and Bryant 2006; Quaedvlieg et al. 2014). The two-locus datasets (ITS and LSU) containing closely related species were used for the analysis. A pairwise homoplasy index lower than 0.05 (Φw < 0.05) indicates significant recombination in the dataset. The relationships among closely related taxa were visualized by constructing split graphs from the concatenated datasets using the LogDet transformation and split decomposition options.

## Results

### Molecular phylogeny

The ITS and LSU dataset (Fig. 1) encompassed 95 fungal specimens representing 81 taxa from 30 families within the order *Agaricales*. Sequences of *Amylocorticium cebennense* (Bourdot) Pouzar were retrieved from GenBank as the outgroup, following a previous study (Dong et al. 2025). Bayesian analysis yielded a topology similar to that of the ML analysis, with an average standard deviation of split frequencies of 0.009685 (BI). The effective sample size (ESS) across the two runs was twice the average ESS (avg ESS), which was 200. The Bayesian analysis results were consistent with the ML tree topology. The phylogenetic tree (Fig. 1) revealed that all species of *Campanella* were grouped within *Campanellaceae*, in which the new species *Ca. albomarginata* C.L. Zhao formed a distinct clade in the genus *Campanella*. Moreover, *Crustomyces*, *Effusomyces*, and *Parvodontia* Hjortstam & Ryvarden were grouped within the family *Cystostereaceae*. The new species *Crustomyces odontoideus* C.L. Zhao was assigned to the genus *Crustomyces* and was closely related to *Cr. tephroleucus* (J. Song, Y.C. Dai & B.K. Cui) Yue Li, Nakasone & S.H. He.

The ITS and LSU dataset (Fig. 2) encompassed 30 fungal specimens representing 16 taxa from the family *Cystostereaceae*. Sequences of *Amylocorticium cebennense* were retrieved from GenBank as the outgroup, following a previous study (Dong et al. 2025). Bayesian analysis yielded a topology similar to that of the ML analysis, with an average standard deviation of split frequencies of 0.009962 (BI). The effective sample size (ESS) across the two runs was twice the average ESS (avg ESS), which was 240.6. In the phylogenetic tree (Fig. 2), the family *Cystostereaceae* included five genera, viz., *Cericium* Hjortstam, *Crustomyces*, *Cystostereum* Pouzar, *Effusomyces*, and *Parvodontia*. The new species *Cr. odontoideus* formed a separate clade, was grouped within the genus *Crustomyces*, and was closely related to *Cr. tephroleucus*.

**Figure 3.**
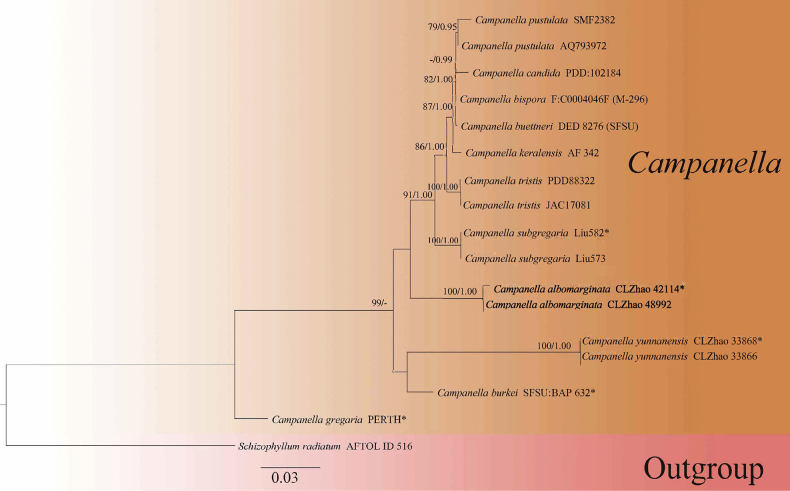
Phylogram generated from a maximum likelihood analysis of *Campanella albomarginata* and related species in *Campanella* based on combined ITS and LSU sequence data; branches are labeled with maximum likelihood bootstrap values ≥ 70% and Bayesian posterior probabilities ≥ 0.95. The new species is in bold.

The ITS and LSU dataset (Fig. 3) encompassed 17 fungal specimens representing 12 taxa of *Campanella*. Sequences of *Amylocorticium cebennense* were retrieved from GenBank as the outgroup, following a previous study (Dong et al. 2025). Bayesian analysis yielded a topology similar to that of the ML analysis, with an average standard deviation of split frequencies of 0.003453 (BI). The effective sample size (ESS) across the two runs was twice the average ESS (avg ESS), which was 320. The phylogeny (Fig. 3) indicated that the new species *Ca. albomarginata* was grouped within the genus *Campanella* and formed an independent lineage.

**Figure 4.**
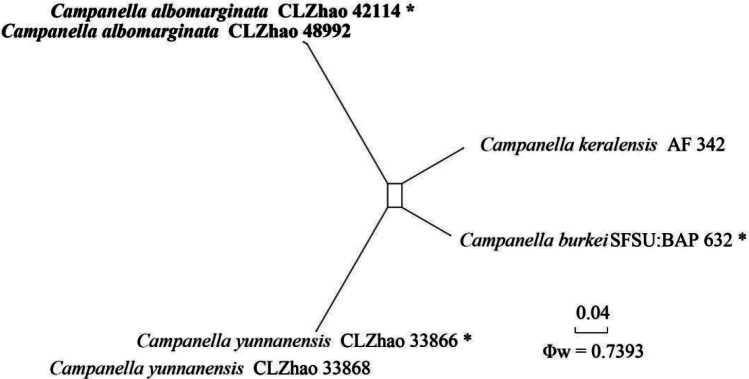
Split graphs showing the results of the PHI test for the ITS and LSU data of *Campanella albomarginata* and closely related taxa using the LogDet transformation and split decomposition. PHI test results of Φw ≤ 0.05 indicate significant recombination within the dataset. The new taxon is in bold. "*" Represents the Holotype.

**Figure 5.**
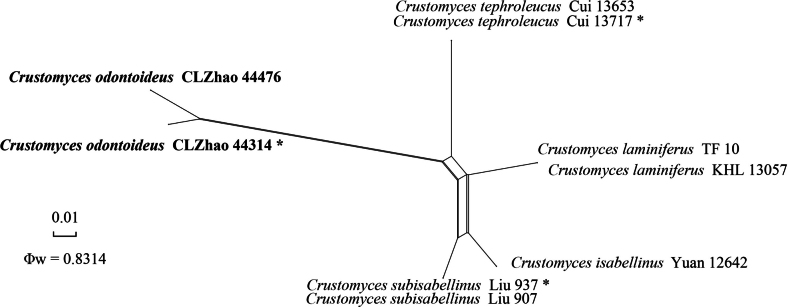
Split graphs showing the results of the PHI test for the ITS and LSU data of *Crustomyces odontoideus* and closely related taxa using the LogDet transformation and split decomposition. PHI test results of Φw ≤ 0.05 indicate significant recombination within the dataset. The new taxon is in bold. "*" Represents the Holotype.

Applying the pairwise homoplasy index (PHI) test to the combined partial ITS and LSU dataset revealed no recombination among phylogenetically related species. No significant recombination events were observed between *Campanella albomarginata* and the phylogenetically closely related species *Ca. burkei*, *Ca. keralensis*, and *Ca. yunnanensis* (Fig. 4). The test results for the combined partial ITS and LSU sequence dataset showed Φw = 0.7393 (Φw > 0.05), indicating that no recombination was present between the new species and *Ca. burkei*, *Ca. keralensis*, or *Ca. yunnanensis*. No significant recombination events were observed between *Crustomyces odontoideus* and the phylogenetically closely related species *Cr. subisabellinus*, *Cr. tephroleucus*, *Cr. laminiferus*, and *Cr. isabellinus* (Fig. 5). The test results for the ITS and LSU sequence dataset showed Φw = 0.8314 (Φw > 0.05), indicating that no recombination was present between the new species and *Cr. subisabellinus*, *Cr. tephroleucus*, *Cr. laminiferus*, or *Cr. isabellinus*.

### Taxonomy

#### Campanella albomarginata

Taxon classificationFungiAgaricalesMarasmiaceae

C.L. Zhao sp. nov.

EDD4F6C8-FA48-5754-9522-E8200B4569C0

863620

Figs 6, 7, 8

##### Etymology.

*albomarginata* (Lat.): referring to the white margin of the type specimen.

**Figure 6.**
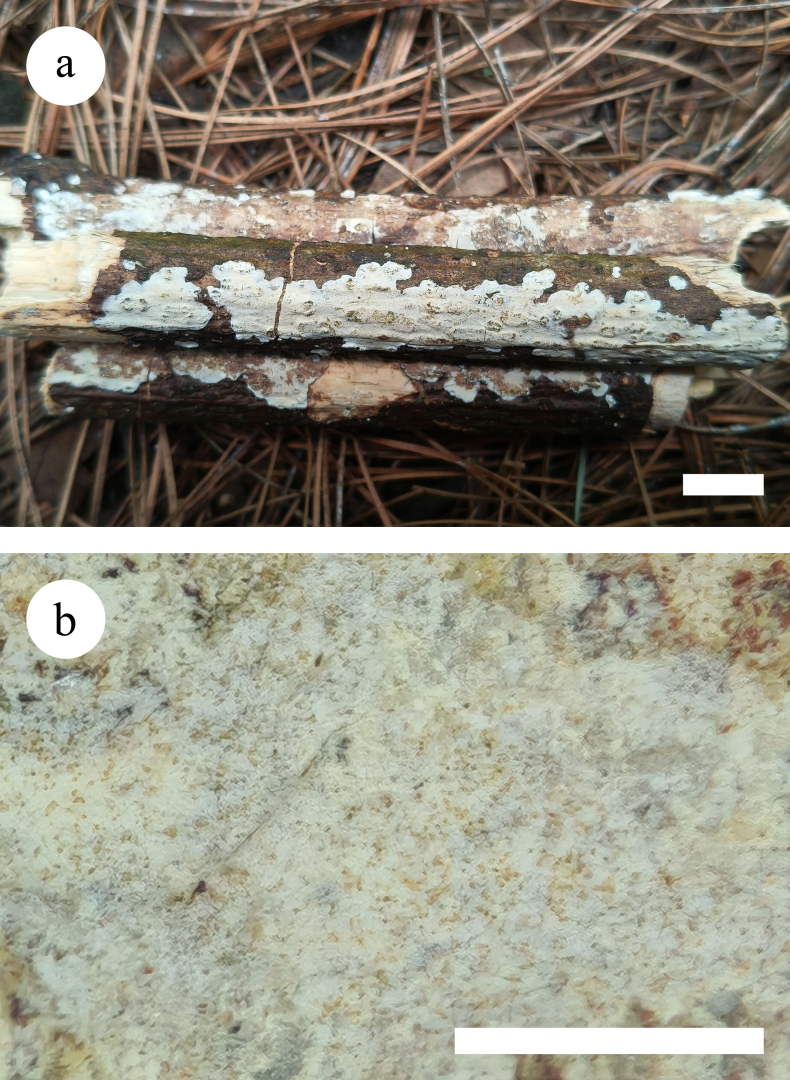
Basidiomata of *Campanella albomarginata* (holotype, CLZhao 42114). **a**. Basidiomata on the substrate; **b**. Hymenophore. Scale bars: 1 cm(**a**); 1 mm (**b**).

**Figure 7.**
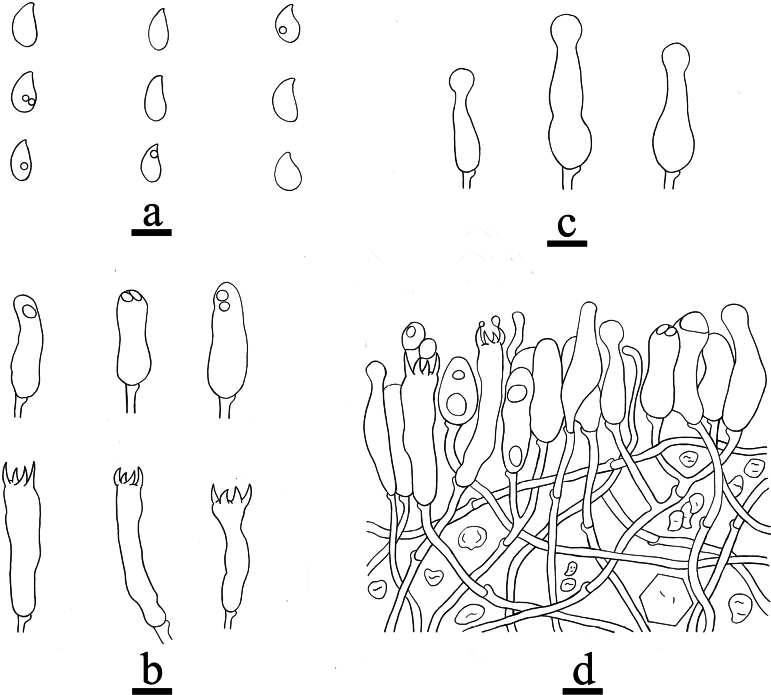
Microscopic structures of *Campanella albomarginata* (holotype, CLZhao 42114). **a**. Basidiospores; **b**. Basidia and basidioles; **c**. Capitate cystidia; **d**. A section of the hymenium. Scale bars: 10 µm (**a–d**).

**Figure 8.**
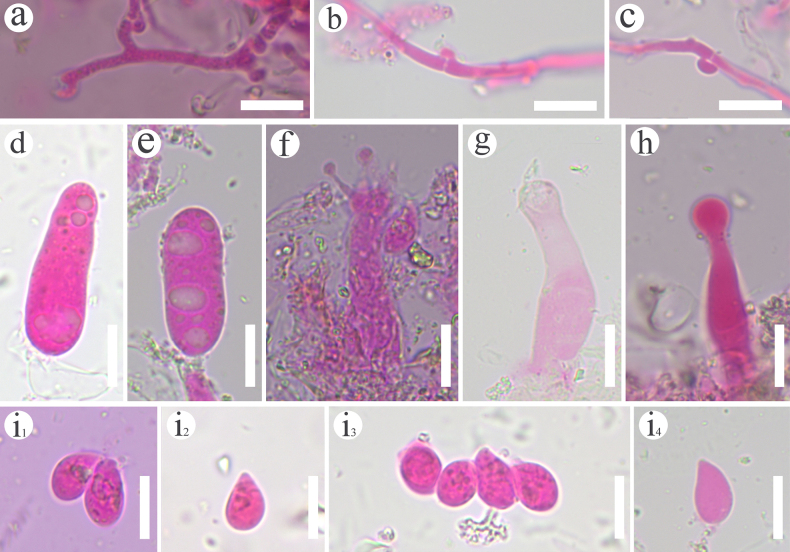
Microscopic structures of *Campanella albomarginata* (holotype, CLZhao 42114). **a–c**. Generative hyphae with clamp connections; **d, e**. Basidioles; **f**. Basidia; **g, h**. Capitate cystidia; **i1–i4**. Basidiospores. Scale bars: 10 µm (**a–i**).

##### Diagnosis.

It is characterized by resupinate, coriaceous basidiomata with white to cream hymenial surface, a monomitic hyphal system with clamped generative hyphae, thin-walled, and drop-shaped basidiospores (9.5–12 × 5–7.5 µm).

##### Holotype.

China, Yunnan Province, Dehong, Ruili, Tongbiguan Provincial Nature Reserve, 24°01'N, 97°83'E, altitude 1000 m asl., on the fallen angiosperm branch, leg. C.L. Zhao, 23 September 2024, CLZhao 42114 (SWFC 00042114).

##### Basidiomata.

Annual, resupinate, coriaceous, without odor or taste when fresh, up to 8 cm long, 1.5 cm wide, up to 200 µm thick. Hymenial surface smooth, white when fresh, turning to cream upon drying. Sterile margin white, thinning out up to 0.5 mm wide.

##### Hyphal system.

Monomitic; generative hyphae with clamp connections, colorless, thin-walled, branched, interwoven, 2–3 µm in diameter; IKI–, CB–; tissues unchanged in KOH. Cystidia capitate, colorless, thin-walled, 29–40 × 6–11 µm; Basidia subcylindrical, slightly constricted, with four sterigmata and a basal clamp connection, 30–47 × 6–10 µm, basidioles dominant, in shape similar to basidia, but slightly smaller. Basidiospores drop-shaped, colorless, thin-walled, smooth, with a few guttulae, IKI–, CB–, (9–)9.5–12(–12.5) × (4.5–)5–7.5(–8) µm, *L* = 10.92 µm, *W* = 6.25 µm, *Q* = 1.40–2.30 (*n* = 60/2).

##### Material examined specimen

**(*paratype*)**. China • Yunnan Province, Dehong, Ruili, Tongbiguan Provincial Nature Reserve, 24°01'N, 97°83'E, altitude 1000 m asl., on the fallen angiosperm branch, leg. C.L. Zhao, 23 September 2024, CLZhao 48992 (SWFC 00048992).

#### Crustomyces odontoideus

Taxon classificationFungiAgaricalesCystostereaceae

C.L. Zhao sp. nov.

7C43AC86-3B34-5EBA-A4FE-793C939439B5

863621

Figs 9, 10, 11

##### Etymology.

*odontoideus* (Lat.): referring to the odontioid hymenial surface of the type specimen.

**Figure 9.**
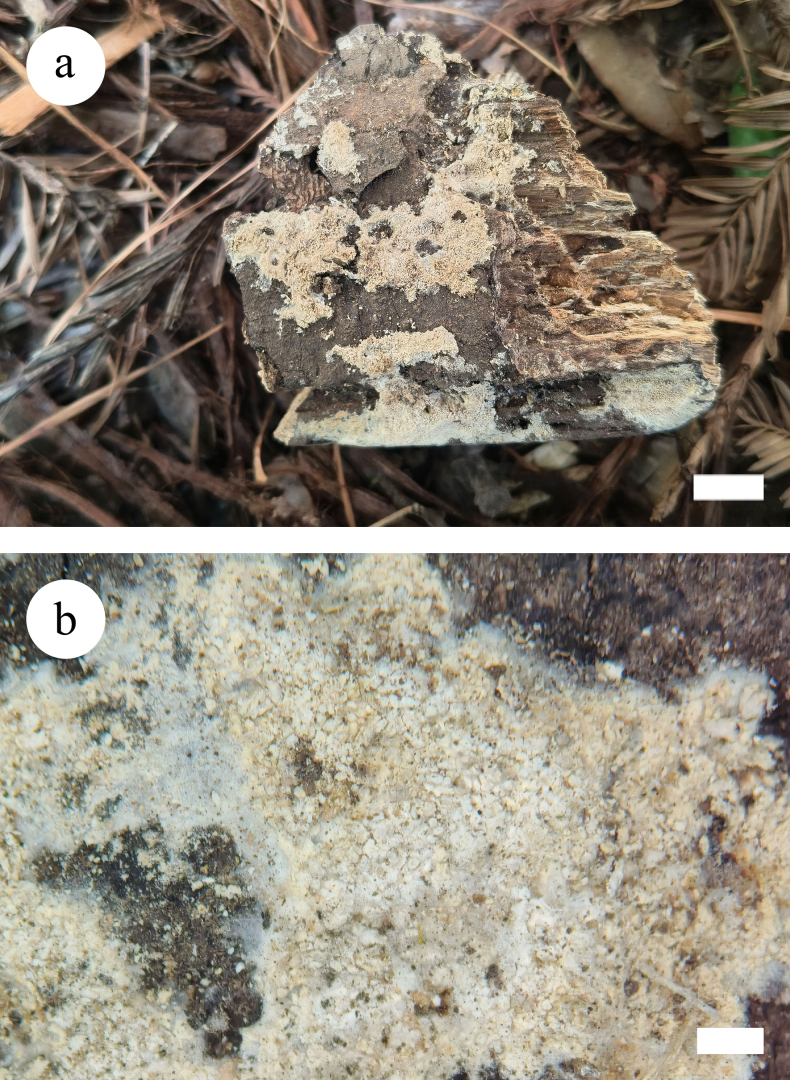
Basidiomata of *Crustomyces odontoideus* (holotype, CLZhao 44314). **a**. Basidiomata on the substrate; **b**. Hymenophore. Scale bars: 1 cm (**a**); 1 mm (**b**).

**Figure 10.**
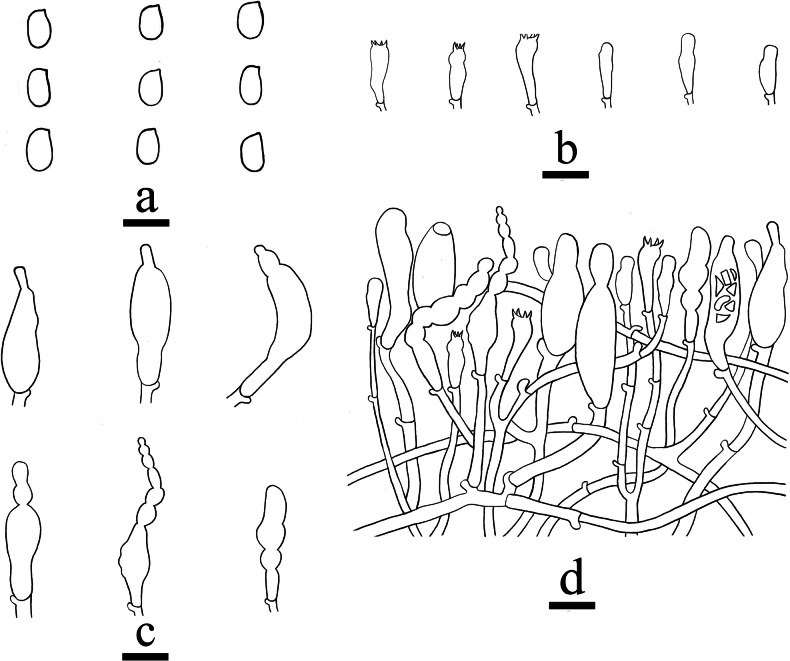
Microscopic structures of *Crustomyces odontoideus* (holotype, CLZhao 44314). **a**. Basidiospores; **b**. Basidioles and basidia; **c**. Gloeocystidia; **d**. A section of the hymenium. Scale bars: 5 µm (**a**); 10 µm (**b–d**).

**Figure 11.**
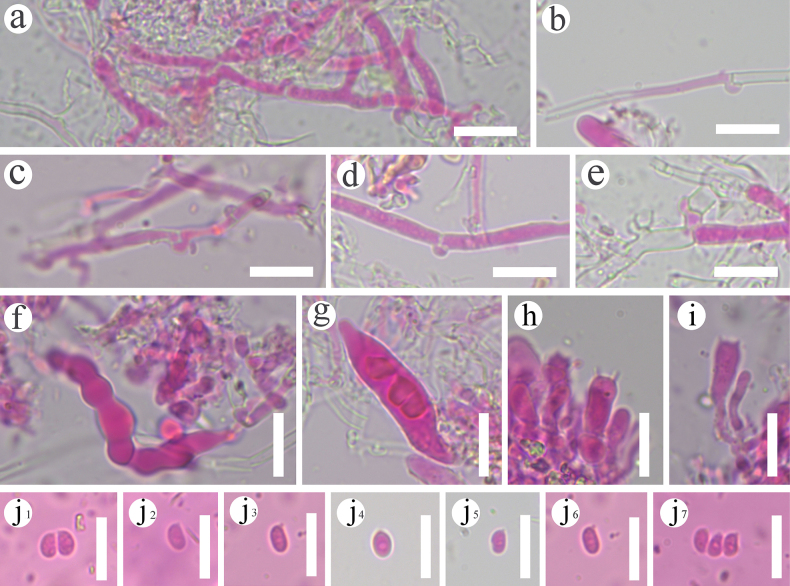
Microscopic structures of *Crustomyces odontoideus* (holotype, CLZhao 44314). **a–e**. Generative hyphae with clamp connections; **f, g**. Gloeocystidia; **h, i**. Basidia; **j1–j7**. Basidiospores. Scale bars: 10 µm (**a–j**).

##### Diagnosis.

It is characterized by odontioid hymenial surface, a monomitic hyphal system with clamped generative hyphae, ovoid to broadly ellipsoid basidiospores (4–4.5 × 2.5–3 µm).

##### Holotype.

China, Yunnan Province, Dehong, Ruili, Tongbiguan Provincial Nature Reserve, 24°01'N, 97°83'E, altitude 1100 m asl., on the fallen angiosperm branch, leg. C.L. Zhao, 15 January 2025, CLZhao 44314 (SWFC 00044314).

##### Basidiomata.

Annual, resupinate, coriaceous, fragile, without odor or taste when fresh, up to 7 cm long, 3 cm wide, up to 100 µm thick. Hymenial surface odontioid, slightly pink when fresh, turning slightly brown upon drying. Sterile margin cream to pink, thinning out up to 0.2 mm wide.

##### Hyphal system.

Monomitic; generative hyphae with clamp connections, colorless, thin-walled, rarely branched, interwoven, 1.5–4 µm in diameter; IKI–, CB–; tissues unchanged in KOH. Gloeocystidia numerous, variable in size and shape, with two types, (1) moniliform, colorless, thin-walled, 29–40 × 6–11 µm; (2) fusiform to subcylindrical, slightly flexuous and inflated, tapering gradually towards the top, 26–57 × 4–12 µm. Basidia subcylindrical to subclavate, with a basal clamp connection and four sterigmata, 11–16 × 3.5–5.5 µm, basidioles dominant, in shape similar to basidia, but slightly smaller. Basidiospores ovoid to broadly ellipsoid, thin-walled, colorless, smooth, IKI–, CB–, 4–4.5(–5) × (2–)2.5–3 µm, *L* = 4.3 µm, *W* = 2.7 µm, *Q* = 1.33–1.91 (*n* = 60/2).

##### Material examined specimen

**(*paratype*)**. China • Yunnan Province, Dehong, Ruili, Tongbiguan Provincial Nature Reserve, 24°01'N, 97°83'E, altitude 1100 m asl., on fallen angiosperm branch, leg. C.L. Zhao, 15 January 2025, CLZhao 44476 (SWFC 00044476).

## Discussion

In the present study, two new species, *Campanella albomarginata* and *Crustomyces odontoideus*, are described based on phylogenetic and morphological analyses.

Several molecular phylogenetic studies have revealed that the genus *Campanella* is monophyletic within the family *Campanellaceae* (Aime and Phillips-Mora 2005; Matheny et al. 2006; Nakasone et al. 2009; Dong et al. 2025). The phylogram based on the combined ITS and LSU sequences (Figs 1, 3) showed that the new species *Ca. albomarginata* was assigned to the genus *Campanella* within the family *Campanellaceae* and was closely related to *Ca. keralensis* Farook & Manim. However, morphologically, *Ca. keralensis* differs from *Ca. albomarginata* in its pleurotoid basidiomata, smaller basidia (27–35 × 7–8 vs. 30–47 × 6–10 µm), longer cheilocystidia (43–56 × 6–9 vs. 29–40 × 6–11 µm), and flexuous-fusoid pleurocystidia (Farook and Manimohan 2014). Morphologically, *Campanella yunnanensis* is similar to *Ca. albomarginata* in having capitate cystidia and drop-shaped basidiospores. However, *Ca. yunnanensis* differs from *Ca. albomarginata* in its longer, capitate cystidia (27.5–35.5 × 5–6.5 vs. 9.5–12 × 5–7.5 µm) and shorter basidiospores (9–11 × 4.5–5.5 vs. 9.5–12 × 5–7.5 µm) (Dong et al. 2025).

Recently, with advances in mycological research, an increasing number of agaric fungi have been recognized as wood-inhabiting fungal species, particularly in many genera within the order *Agaricales*, such as *Mycena* (Pers.) Roussel, *Marasmius* Fr., and *Resupinatus* Nees ex Gray (Dong et al. 2025; Zhuang et al. 2026). Similarly, *Marasmius*, a member of the mushroom-forming fungi and a highly morphologically diverse genus in *Agaricales* (Bhunjun et al. 2022), includes the type species *M. rotula* (Scop.) Fr., which has a typical agaricoid form with a pileus, stipe, and lamellae, whereas the newly described *M. wumengshanensis* J.H. Dong & C.L. Zhao is resupinate, lacking both a pileus and stipe (Dong et al. 2025). Furthermore, species of *Resupinatus* Nees ex Gray are widely distributed worldwide and are characterized by small, pleurotoid to cupulate-lamellate basidiomata; cyphelloid, poroid, and merulioid forms are also included in the genus (Carpouron et al. 2024), whereas some species have resupinate basidiomata, namely, *R. angulatus* Q.Xin Guan, H. Zhao, L. Zhuang, Yuan Yuan & Y.C. Dai, *R. latemarginatus* Q.Xin Guan, H. Zhao, L. Zhuang, Yuan Yuan & Y.C. Dai, and *R. sinuosus* Q.Xin Guan, H. Zhao, L. Zhuang, Yuan Yuan & Y.C. Dai (Zhuang et al. 2026). Notably, significant morphological divergence within a single genus is common in *Agaricales*. Nevertheless, only *Ca. albomarginata* and *Ca. yunnanensis* have resupinate basidiomata, whereas all remaining species classified under *Campanella* have pileate basidiomata. This morphological divergence within the genus requires further taxonomic discussion.

*Cystostereaceae* is a small family in the order *Agaricales* that includes seven genera, viz., *Cystostereum*, *Crustomyces*, *Cericium*, *Cystidiodontia*, *Parvobasidium*, *Parvodontia*, and *Rigidotubus* (Jülich 1978, 1981; Larsson 2007). Based on the combined ITS and LSU sequences (Fig. 1), the phylogram showed that *Crustomyces odontoideus* was assigned to the genus *Crustomyces*, in which *Cr. odontoideus* formed an independent lineage. In addition, it was closely related to *Cr. tephroleucus*, with support values of 100/1.00 in Fig. 1 and 84/– in Fig. 2. However, morphologically, *Cr. tephroleucus* differs from *Cr. odontoideus* in having tough, poroid basidiomata, a white to grayish-white pore surface, and shorter clavate basidia (8–12 × 3–5 vs. 11–16 × 3.5–5.5 µm; Song et al. 2018; Li et al. 2022). Morphologically, *Crustomyces albidus* is similar to *Cr. odontoideus* in having similar moniliform gloeocystidia, clavate basidia, and generative hyphae with clamp connections. However, *Cr. albidus* differs significantly in having a white, smooth hymenophore and larger basidia (14–22 × 4–5.5 vs. 11–16 × 3.5–5.5 µm; Li et al. 2022). In addition, *Cr. odontoideus* is similar to *Cr. isabellinus*, *Cr. laminiferus*, and *Cr. subisabellinus* in having resupinate to effused basidiomata and clamped generative hyphae. However, *Cr. isabellinus* differs from *Cr. odontoideus* in having distinctly dextrinoid and cyanophilous hyphal walls and smaller basidiospores (3–4 × 2.5–3 vs. 4–4.5 × 2.5–3 µm; Hallenberg and Ryvarden 1975); *Cr. laminiferus* differs from *Cr. odontoideus* in having a variably shaped pileal margin that is hydnoid to subporoid, a grayish-brown pileus, a dimitic hyphal system, and smaller basidiospores (3–3.2 × 2.5–2.8 vs. 4–4.5 × 2.5–3 µm; Hjortstam and Ryvarden 1986); and *Cr. subisabellinus* differs from *Cr. odontoideus* in its dimitic hyphal system, fusoid to narrowly clavate gloeocystidia, and smaller gloeocystidia (15.5–20.3 × 4.2–5.7 vs. 29–40 × 6–11 µm; Liu et al. 2025).

Yunnan Province, with its highly diverse ecological environments, harbors a wide range of organisms, including animals, plants, and fungi (Liu et al. 2025; Wang et al. 2023; Yang et al. 2025; Zheng et al. 2026). Given this remarkable biodiversity, it is likely that numerous species remain undocumented (Zhao et al. 2024). Consequently, further systematic surveys are essential to characterize the diversity and distribution of *Agaricales* more comprehensively. This study provides information on two new *Agaricales* species, thereby enriching the current mycological research dataset on *Campanella* and *Crustomyces* and providing evidence for future taxonomic, phylogenetic, and distributional studies.

## Supplementary Material

XML Treatment for Campanella albomarginata

XML Treatment for Crustomyces odontoideus

## Additional information

### Artificial Intelligence (AI) use

The authors accept full responsibility for the content of the manuscript, including the disclosure of any use of AI.

No AI tools were used in the preparation of this manuscript.

### Author ORCIDs

W. Liu https://orcid.org/0009-0008-6281-8904

Y. Deng https://orcid.org/0000-0002-8220-508X

X. Liu https://orcid.org/0000-0003-0100-2094

C. Zhao https://orcid.org/0000-0002-8668-1075

### Data availability

All of the data that support the findings of this study are available in the main text or Supplementary Information.

## Supplementary materials

Supplementary material 1 Sequence of Figure 1 Data type fasta https://binary.pensoft.net/file/1737009This dataset is made available under the Open Database License (http://opendatacommons.org/licenses/odbl/1.0/). The Open Database License (ODbL) is a license agreement intended to allow users to freely share, modify, and use this Dataset while maintaining this same freedom for others, provided that the original source and author(s) are credited. Wanting Liu, Yinglian Deng, Xiangfu Liu, Changlin Zhao

Supplementary material 2 Sequence of Figure 2 Data type fasta https://binary.pensoft.net/file/1737010This dataset is made available under the Open Database License (http://opendatacommons.org/licenses/odbl/1.0/). The Open Database License (ODbL) is a license agreement intended to allow users to freely share, modify, and use this Dataset while maintaining this same freedom for others, provided that the original source and author(s) are credited. Wanting Liu, Yinglian Deng, Xiangfu Liu, Changlin Zhao

Supplementary material 3 Sequence of Figure 3 Data type fasta https://binary.pensoft.net/file/1737011This dataset is made available under the Open Database License (http://opendatacommons.org/licenses/odbl/1.0/). The Open Database License (ODbL) is a license agreement intended to allow users to freely share, modify, and use this Dataset while maintaining this same freedom for others, provided that the original source and author(s) are credited. Wanting Liu, Yinglian Deng, Xiangfu Liu, Changlin Zhao

Supplementary material 4 Sequence of Figure 4 Data type fasta https://binary.pensoft.net/file/1737012This dataset is made available under the Open Database License (http://opendatacommons.org/licenses/odbl/1.0/). The Open Database License (ODbL) is a license agreement intended to allow users to freely share, modify, and use this Dataset while maintaining this same freedom for others, provided that the original source and author(s) are credited. Wanting Liu, Yinglian Deng, Xiangfu Liu, Changlin Zhao
